# Prevalence and Associated Factors of Poststroke Depression among Outpatient Stroke Patients Who Have a Follow-Up at the Outpatient Neurology Clinic of Zewditu Memorial Hospital in Addis Ababa, Ethiopia

**DOI:** 10.1155/2022/9750035

**Published:** 2022-03-22

**Authors:** Tsion Yehualashet Wubshet, Sisay Gizaw Geberemichael, Takle Menna Adilo, Temesgen Tantu Arusi, Muluken Gunta Gutulo, Dereje Zewdu Assefa, Mekete Wondesen Asfaw

**Affiliations:** ^1^St. Paul's Hospital Millennium Medical College, Addis Ababa, Ethiopia; ^2^Wolkite University College of Medicine and Health Sciences, Ethiopia; ^3^Wolaita Zone Health Department, Ethiopia

## Abstract

**Background:**

Poststroke depression is the most common and burdensome poststroke psychiatric complication. Studies showed discrepancies in reporting frequencies and risk factors for poststroke depression. Updated local data are relevant for efficient strategies of poststroke depression screening and prevention.

**Objectives:**

To determine the prevalence and associated factors of poststroke depression among outpatient stroke patients from the outpatient neurology clinic of Zewditu Memorial Hospital in Addis Ababa, Ethiopia.

**Methods:**

An institution-based cross-sectional study was conducted on 249 stroke patients. Data was collected through structured questionnaire using interviews and a review of medical charts. PHQ-9 depression questionnaire was used to diagnose poststroke depression. Descriptive analysis was used to see the nature of the characteristics of interests. Bivariate analysis was used to sort out variables at *p* values less than 0.05 for multivariate logistic regression. Significance level was obtained using an odds ratio with 95% CI and *p* value < 0.05.

**Results:**

Point prevalence for poststroke depression was 27.5 percent. Female gender, unemployment, low social support level, diabetes mellitus, and poststroke period under 2 years were statistically significant and independent predictors for poststroke depression.

**Conclusions:**

The point prevalence estimate of poststroke depression was comparable with other studies. Low social support levels increased the odds for poststroke depression by more than eight folds. It appeared that external factors are more important in the pathogenesis of poststroke depression in the African population. Detection and prevention programs should consider disparities of poststroke depression incidence and risk factors.

## 1. Introduction

Stroke may cause several mood-related psychiatric syndromes including depression, anxiety, and adjustment disorder [1]. Depression is the commonest of all poststroke mood and neuropsychiatric disorders [1, 2]. Poststroke depression (PSD) includes a range of clinical severity and can be classified as early onset (within 6 months) or late onset (beyond 6 months) based on the time since stroke [2, 3]. PSD may coexist or overlap with clinical features of other poststroke mood disorders. Furthermore, clinical diagnosis of PSD is perplexing as some stroke manifestations may mimic or mask clinical features of PSD. Stroke-related dysphasia poses particular challenges for PSD screening and diagnosis. However, PSD clinical assessment and evaluation have been facilitated by standardized clinical tools and diagnostic criteria [1–4].

PSD is commonly defined and classified by diagnostic criteria in the Diagnostic and Statistical Manual of Mental Disorders fifth edition (DSM-V) and International Classification of Diseases (ICD). Several structured questionnaires such as Center of Epidemiological Studies-Depression Scale (CES-D), Hamilton Depression Rating Scale (HDRS), Patient Health Questionnaire item 9 (PHQ-9), Montgomery-Asberg Depression Rating Scale (MADRS), DSM-V Depressive Ideation Assessment (DIA), Geriatric depression scale (GSI), community-based rehabilitation indicators (CBH), and Hospital Anxiety and Depression Scale (HADS-Total and HADS-D) are available for clinical assessment of PSD. The structured questionnaires are validated rating scales for subjective depressive symptoms and provide data for diagnostic classification of PSD [2, 5–11].

In global estimates, one-fourth to one-third of stroke survivors develop PSD. The reported cumulative prevalence of PSD is 55% in one systematic review reporting global PSD epidemiology. PSD is reported in 31-33% of stroke survivors in developed and western societies [2, 5, 12, 13]. In Africa, PSD prevalence ranges from 22.9 to 53.6%. Pooled prevalence estimate in sub-Saharan Africa (SSA) was 31%. PSD is thus one of the major public health concerns in nearly all regions of the world; although, population prevalence varies widely among different geographic locations [14–18].

PSD point prevalence varies widely in different study settings and with different measurement tools. Generally, community-based studies reported lower PSD prevalence (9-14%) than studies in stroke hospitals and services (20-36%) [2, 8, 13]. PSD prevalence is highest in acute and immediate postacute poststroke periods and at inpatient stroke services (32-36%) than during long-term stroke follow-up at outpatient settings (23.9-24%). PSD risk factors may differ in prevalence along the course of stroke and are likely to contribute to the observed difference in PSD prevalence along the spectrum of the poststroke continuum of care at various settings [2, 4, 13, 15].

Various factors like age, gender, psychiatric illness, family history of psychiatric illness, hypertension, diabetes mellitus (DM), angina pectoris, current cigarette smokers, low socioeconomic status, low monthly income, unemployment, marital status, and educational background as well as the involvement of the left hemisphere of the brain of the patient are associated with PSD [2–5, 7, 12–21]. Most of these prestroke and poststroke risk factors reduce the potential to cope with disability and are more relevant predictors of PSD in developing countries of SSA.

In SSA, stroke location and stroke type appear to have a lesser influence on PSD incidence [16, 22, 23]. Population prevalence for depression in Ethiopia is estimated to be 6.5% (24). Depressive mood disorders are important contributors to increasing mental illness burden and healthcare costs. Undiagnosed and untreated depression poses huge direct and indirect healthcare costs to the country and societal challenges. The study addressed PSD in Ethiopia even though both stroke and depression are common morbidities in the country. To the authors' knowledge, this study is the first of its kind that determines the epidemiology of PSD in Ethiopia.

## 2. Methods

### 2.1. Study Area, Study Setting, and Study Design

A hospital-based cross-sectional study was conducted at the Neurology Department of Zewditu Memorial Hospital (ZMH) in Addis Ababa, Ethiopia, from April 1 to August 30, 2020. All clients who have a history of stroke and follow-up at the Outpatient Neurology Department of ZMH (ONC-ZMH) are the source population. On average, 520 clients (new and follow-ups) attend the clinic of the Outpatient Neurology Department every month (estimated by the authors from health management information system (HMIS) records of ZMH). But the above report was based on previous data which is before the emergence of the COVID-19 pandemic; as a result, there was a significant reduction in the number of clients visiting the hospital.

### 2.2. Subject Selection

The sample size was calculated using a formula for a single population proportion. Since no study was done, 50 percent PSD prevalence was assumed in the study population at 0.05 error margin (significance level) and 95 percent confidence interval values. Calculated sample size after adjusting for short study duration and including 10 percent estimated nonresponse rate was 249. Study samples were selected by using a systematic random sampling technique from the HMIS register of ONC-ZMH which was the sampling frame. After the first sample, the total population was divided to sample sizes to get the interval (*k*). On each day of the study period, the first sample was identified randomly; then, after, every *k* patient was selected.

Nonresponders include those who did not consent or those who had communication problems (such as aphasia) or those receiving drugs (esp. antidepressants) or had underlying comorbidities that may modify the primary dependent variable of interest in the study.

### 2.3. Study Instruments

A structured (interviewer-assisted administration) questionnaire was used in the study. The questionnaire was developed following standard recommendations to meet specific needs and contexts of the study. A thirty-nine-item questionnaire was constructed in the English language with extensive review of relevant literature and prior PSD questionnaires. The English questionnaire version was translated and retranslated back into the local language (Amharic) by the professionals. The questionnaire was pretested on twenty-five samples of clients from a neurology clinic of Black Lion Tertiary (university) Hospital in Addis Ababa then modified based on the feedback. The data collection tools contain sociodemographic and clinical variables. Diagnosis of PSD was made by questionnaires adopted from Patient Health Questionnaire version nine (PHQ-9) and DSM-V TR.

### 2.4. Data Collection Procedures

Five interviewers (interns and nurses) and a supervisor (intern) were recruited for the study. The principal investigator trained interviewers and supervisors for three days using a training manual. Trainees were overviewed about the purpose of the study, components of the thirty-nine-item study questionnaire, and standards for interviewer-assisted questionnaire administration. Demonstration and dummy practice on questionnaire administration, face-to-face interview procedures, and review of clients' charts were performed to complement the interview. Sampled clients were invited to participate in the study after they received their medical service. Exit face-to-face interview was done on those who consented, and their medical charts were reviewed to complete the missing information in the questionnaire. The supervisor monitored data collection processes and checked the questionnaire for completeness and was involved in sampling of clients and arrangement of a private interview.

### 2.5. Statistical Analysis

Items in sections 4 and 5 were scored and summarized to facilitate analysis. Twelve items in section 4 formed three separate subscales for measuring perceived social support from significant others, friends, and families during the current stroke. Seven-level Likert scale responses for the twelve variables were converted to numbers [1–7]. Responses for four items in each subscale were averaged (each response summed up and divided by four) to get the mean subscale total score. Mean subscale total score was categorized as low social support (1-2.9), moderate social support (3–5), and high social support (5.1-7). Similarly, responses for items in section-5 (categorical nominal or ordinal variables) were summarized based on mean section total score (by assigning 1 for yes response and 0 for no response or 0 to 3 for PHQ-9). Accordingly, the mean section total score was categorized as no depression, minor depression, and major depression; or no depression (0-4) mild depression [5–9], moderate depression [10–14], moderately severe depression [15–19], and severe depression [20–26].

Data from the main study were entered and analyzed using SPSS for Windows version 25.0 software. Frequencies and proportions described categorical data (nominal variables and summarized (converted) ordinal variables). Crosstabulation and chisquared tests were used to assess the association of PSD with different factors including sociodemographic and stroke characteristics, medical comorbidities, and social support levels. In the chi-squared testing, chi-squared values were interpreted along with the degree of freedom (df) and 0.05 level of significance.

Logistic regression analyses further explored degrees and directions of association of factors with PSD (identified by chi-squared tests) and clarified possible confounding effects of associations between factors and PSD. In all unmatched logistic regression analyses, regression coefficient, adjusted odds ratio (AORs), and 95 percent confidence interval (CI) were reported and interpreted at 0.05 (*p* value) significance level.

Stroke subjects were dichotomized into those with depression and those for comparative analysis. Means and medians were compared using the Student *t*-test or Mann–Whitney *U*-test for paired comparisons. Proportions were compared using the chi-squared test with Yates correction for proportions with subgroupings < 5. A multivariate logistic regression analysis was performed to identify independent predictors of depression. In all analyses, two-tailed *p* values < 0.05 were considered statistically significant with no adjustments for multiple comparisons. Statistical analysis was performed using SPSS version 19.

## 3. Results

### 3.1. Sociodemographic Results

**Figure 1:**
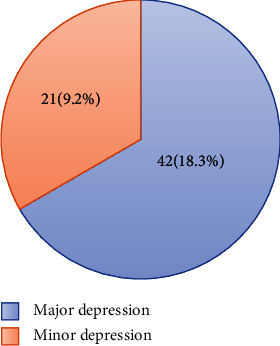
Distribution of major and minor depression on study respondents at the neurology clinic of Zewditu Memorial Hospital in Addis Ababa, Ethiopia (*n* = 63).

A total of 229 patients agreed and responded from the total sample size which makes the response rate 92%. Sixty-three respondents (27.5%) had PSD. Out of these PSD patients, 42 (18.3%) had major depression and 21 (9.2%) had minor depression (Figure 1). There are 138 male patients, and the rest are female. Most of the patients are in the age range of 45-74 years old. (Table 1)

### 3.2. Clinical and Anatomic Characteristics of the Patients

Most of the patients have sustained ischemic stroke (131), and its location is on the left hemisphere (118). Fifty-eight patients presented within the first 24 months of the insult. Of 58 patients, 15 patients have depression (Table 2).

### 3.3. Factors Associated with PSD

The following factors are associated with the prevalence of PSD. In binary logistic analysis, factors like living alone, DM, employment status, sex, poststroke duration < 24 months, and having social support were associated with PSD. Those variables which have an association in bivariate logistic regression were further analyzed with multivariate logistic analysis. Only sex, poststroke duration < 24 months and having social support were strongly associated with PSD during multivariate logistic analysis (Table 3).

## 4. Discussion

Our study showed a 27.5 percent prevalence of PSD in the population of stroke patients. PSD morbidity burden was segregated according to severity; the study documented prevalence figures of 18.3 and 9.2 percent for major PSD and minor PSD, respectively. This prevalence rate is similar to the pooled prevalence of PSD in systemic reviews and most prevalence figures in developed countries [1, 3, 19]. However, our study showed a lower prevalence of PSD compared to most studies in Africa and developing countries in Asia [5, 15, 18, 20, 25]. Several factors may contribute to these apparent differences in population estimates for PSD prevalence. One possible reason is sociocultural differences and the effects of social support networks. The difference in methodology and sample sizes may also contribute to the observed variations in PSD prevalence but the prevalence is higher than in other African studies [17, 26]. Moreover, it is of note that depression is threefold more common among the study population than in the general adult population of the country [24].

In our study, consistent with most studies, the female gender is found to be an independent predictor for PSD. In our study, females have increased odds of having PSD compared to males. Female gender was associated with depression in epidemiological studies of depression in the general population in Ethiopia [13, 22, 24]. Our study is also consistent with most studies in Africa and elsewhere in showing female sex as a risk factor for PSD [13, 16, 20, 26]. Only a few studies reported male gender as an independent predictor for PSD (28). Some studies showed no association with gender [21]. PSD is consistently linked with low social support levels. Psychosocial difficulties are generally considered as a risk factor for the onset of PSD [3, 17]. More than one-third of the study subjects were at or below medium social support status. Low social support status was found statistically associated with PSD. Similar observations highlighting the role of social support in the development of PSD have been stated in several reports [3, 15, 16, 28].

Similarly, unemployment is associated with increased risk for PSD in our study which goes with other studies [14]. Stroke survivors with depression are more prone to financial challenges because of unemployment, increased health care bills, and a virtual lack of insurance coverage. This has a spiral negative impact on family support systems that shoulder health care cost burden and caregiving. All these factors are interlinked and produce a synergy effect in causing poststroke depression when combined in a stroke patient [15, 17].

Our study showed increased odds of PSD during the first year poststroke period. This finding is in congruence with other studies from China and India. In other studies, PSD was more prevalent in early periods of poststroke with an increased tendency for being chronic [3, 16, 23]. Early-onset PSD is likely to be related to multiple biopsychosocial factors in its generation and persistence [3, 5, 23].

The link between stroke lesions and incidence of PSD is inconsistent and appears irrelevant in African settings. Stroke localization and stroke subtypes were not associated with PSD in this study. The African studies conducted in Congo, Nigeria, Egypt, and Ghana had the same results [3–5, 7, 16]. The studies from India and China, which only assessed patients in the early few months after stroke, had different results [16, 23]. This was evidenced by the tendency of depression to associate more with reduced social and economic potential to cope with disability than the biological mechanism like stroke type and location in African patients [18].

## 5. Conclusions

It was concluded that the prevalence of poststroke depression is high and remained underrecognized. Low social support status, female sex, and unemployed patients were more vulnerable to develop poststroke depression. Those who usually have low social support status and at the first year of poststroke depression were at higher risk of developing depression after stroke.

## Figures and Tables

**Table 1:** Distribution and chi-squared tests of PSD in relation to some sociodemographic characteristics of study respondents at the neurology clinic of Zewditu Memorial Hospital in Addis Ababa, Ethiopia (total *N* = 229).

Sociodemographic characteristic	Number *N* (%)	Depressed *N* (%)	Nondepressed *N* (%)	*p* value
Gender				
Male	138 (60.3)	28 (20.28)	110 (79.7)	0.003
Female	91 (39.7)	35 (38.46)	56 (61.5)	
Age group (years)				
<45	34 (14.8)	10 (29.4)	24 (70.6)	0.708
45-60	86 (37.6)	26 (30.2)	60 (69.8)	
61-74	87 (38.0)	23 (26.4)	64 (73.6)	
>75	22 (9.6)	4 (18.2)	18 (81.8)	
Residence				
Urban dwellers	201 (87.8)	55 (27.4)	146 (72.6)	0.893
Rural dwellers	28 (12.2)	8 (28.6)	20 (71.4)	
Employment status				
Employed	51 (22.3)	7 (13.7)	44 (86.3)	0.001
Unemployed	129 (56.3)	48 (37.2)	81 (62.8)	
Retired	49 (21.4)	8 (16.3)	41 (83.7)	
Education level				0.654
Illiterate	24 (10.5)	1 (4.1)	17 (70.8)	
Can read & write	16 (7.0)	6 (37.5)	11 (68.75)	
Primary school	63 (27.5)	13 (20.6)	50 (79.4)	
Secondary school	113 (49.3)	35 (30.97)	78 (69.02)	
Higher education	13 (5.7)	3 (23.07)	10 (76.9)	
Social support level				<0.01
High	144 (62.9)	18 (12.5)	126 (87.5)	
Low and medium	85 (33.2)	45 (52.9)	40 (47)	
Living alone	22 (9.6)	12 (54.5)	10 (45.5)	

**Table 2:** Distribution of PSD in relation to some clinical and anatomic characteristics of stroke in study respondents at the neurology clinic of Zewditu Memorial Hospital in Addis Ababa, Ethiopia (total *N* = 229).

Variables	Number *N* (%)	Depressed *N* (%)	Nondepressed *N* (%)	*p* value
Stroke type				
Ischemic	131 (57.2)	42 (32.1)	89 (67.9)	0.075
Haemorrhagic	98 (42.8)	(21.4)	(78.6)	
Stroke location				
Left hemispheric	118 (51.5)	33 (28.0)	85 (72.0)	0.570
Right hemispheric	107 (46.7)	28 (26.2)	79 (73.8)	
Bilateral	4 (1.7)	2 (50)	2 (50)	
Stroke duration				0.01
24 months	58 (25.3)	15 (25.9)	43 (74.1)	
24 months	78 (34.1)	11 (14.1)	67 (85.9)	
Stroke risk factor				
Hypertension	161 (70.3)	47 (29.2)	114 (70.8)	
Diabetes mellitus	47 (20.5)	19 (40.4)	28 (59.6)	
Smoking	48 (21.0)	13 (27.08)	35 (72.9)	
Prior stroke	22 (9.6)	(22.7)	(77.3)	
Coronary artery disease	2 (0.9)	1 (50%)	1 (50%)	
Prestroke psychiatric illness	0			
Depression family history				
Positive	6 (2.6)	3 (50.0)	3 (50.0)	0.211
Negative	223 (97.4)	60 (26.9)	163 (73.09)	

**Table 3:** Bivariate and multivariate logistic regression of factors associated with PSD at the outpatient neurology clinic of Zewditu Memorial Hospital in Addis Ababa, Ethiopia (total *N* = 229).

Variables	Categories	Depressed *N* (%)	Nondepressed *N* (%)	Crude odds ratio 95% CI	Adjusted odds ratio 95% CI
Gender	Male	28 (20.28)	110 (79.7)	0.407 (0.225-0.736)	0.425 (0.1919, 0.944)^∗^
	Female	35 (38.46)	56 (61.5)	1.0	
Employment status	Employed	7 (13.7)	44 (86.3)	0.85 (1.223-6.805)	0.938 (0.266, 3.312)
	Unemployed	48 (37.2)	81 (62.8)	3.037 (1.314-7.017)	2.875 (1.001-8.189)^∗∗^
	Retired	8 (16.3)	41 (83.7)	1.0	
DM	Yes	19 (40.4)	28 (59.6)	2.128 (1.084-4.177)	2.14 (0.889-5.088)
	No	44 (24.1)	138 (75.9)	1.0	
Post stroke duration	≤24 months	15 (25.9)	43 (74.1)	3.199 (1.556-6.576)	5.34 (2.157-13.285)^∗∗^
	>24 months	11 (14.1)	67 (85.9)	1.0	
Living alone	Yes	12 (54.5)	10 (45.5)	3.671 (1.497-8.999)	1.782 (0.620-5.119)
	No	51 (24.6)	156 (75.4)	1.0	
Social support	High support	18 (12.5)	126 (87.5)	1.0	
	Low & moderate support	45 (52.9)	40 (47)	7.875 (4.102-15.117)	11.6 (5.040-25.153)^∗∗^

^∗^ *p* < 0.05; ^∗∗^*p* < 0.005.

## Data Availability

It is available on request from the corresponding author.

## Acronyms

ZMH: Zewditu Memorial Hospital
PSD: Poststroke depression
SPHMMC: Saint Paul's Hospital Millennium Medical College
DM: Diabetes mellitus
ONC: Outpatient Neurology Clinic.

## Additional Points

*Limitations*. Being a cross-sectional study, this study inherits in itself the limitation to test the temporal sequence of these events and interpretation for causal inferences. The study was limited to one public sector hospital. Our entire sample was based on those who had no communication difficulty and other chronic CNS conditions.
